# A Multiple Target Positioning and Tracking System Behind Brick-Concrete Walls Using Multiple Monostatic IR-UWB Radars

**DOI:** 10.3390/s19184033

**Published:** 2019-09-18

**Authors:** Sungwon Yoo, Dingyang Wang, Dong-Min Seol, Chulsoo Lee, Sungmoon Chung, Sung Ho Cho

**Affiliations:** 1 Department of Electronics and Computer Engineering, Hanyang University, Seoul 04763, Korea; irishtaco@hanyang.ac.kr (S.Y.); wdyggh@hanyang.ac.kr (D.W.); 2 Data Link & Positioning System, LIGNex1 Co., Ltd., Seongnam 13488, Korea; dongmin.seol@lignex1.com (D.-M.S.); chulsoo.lee@lignex1.com (C.L.); 3 Defense Industry Technology Center, Agency of Defense Development, Seoul 04353, Korea; dearsm@add.re.kr

**Keywords:** impulse radio ultra-wideband radar, through-wall radar imaging, multiple target positioning and tracking, wall characteristic estimation, constant false alarm rate, positioning algorithm

## Abstract

Recognizing and tracking the targets located behind walls through impulse radio ultra-wideband (IR-UWB) radar provides a significant advantage, as the characteristics of the IR-UWB radar signal enable it to penetrate obstacles. In this study, we design a through-wall radar system to estimate and track multiple targets behind a wall. The radar signal received through the wall experiences distortion, such as attenuation and delay, and the characteristics of the wall are estimated to compensate the distance error. In addition, unlike general cases, it is difficult to maintain a high detection rate and low false alarm rate in this through-wall radar application due to the attenuation and distortion caused by the wall. In particular, the generally used delay-and-sum algorithm is significantly affected by the motion of targets and distortion caused by the wall, rendering it difficult to obtain a good performance. Thus, we propose a novel method, which calculates the likelihood that a target exists in a certain location through a detection process. Unlike the delay-and-sum algorithm, this method does not use the radar signal directly. Simulations and experiments are conducted in different cases to show the validity of our through-wall radar system. The results obtained by using the proposed algorithm as well as delay-and-sum and trilateration are compared in terms of the detection rate, false alarm rate, and positioning error.

## 1. Introduction

In recent times, impulse radio ultra-wideband (IR-UWB) radar has been highly and actively used in many areas, such as human detection, through-wall imaging, people counting, and indoor positioning [1,2,3,4,5,6,7,8]. Employing the IR-UWB radar offers many advantages. This radar exhibits a high time resolution by exploiting a wide frequency area, which enables precise distance measurements. Occupying a wide frequency area also allows it to penetrate obstacles and remain robust to changes in the environment. In addition, the IR-UWB radar emits Gaussian pulses, which results in very low duty cycle and a low power consumption.

Applications related to obstacle penetration are areas that are being studied very actively and can be useful in military operations, intrusion detection, and rescuing people during times of disaster [9,10,11,12,13,14,15,16]. In particular, the requirement for such applications in the military field is developing rapidly, as recent military operations are frequently conducted in the city. It is not easy to recognize these situations in the indoor environment due to obstacles, such as walls. The IR-UWB radar is beneficial for obstacle penetration and remains robust to ambient disturbance due to its characteristics. Therefore, it generates a high demand in the military area.

Various studies have been conducted to recognize situations behind walls by penetrating these walls with radars, many of which have focused on devising methods to create the radar images [17,18,19]. However, the techniques for estimating and tracking the location of multiple targets from these radar images have not been studied much, as estimating and tracking the location of multiple targets from radar images requires the target signal to be maintained high according to the change of targets’ radar cross section. Several studies have been conducted to estimate and track multiple targets behind the wall using different methods. In [20], the time of arrival (TOA) between the radar and target is estimated, and the locations of multiple targets are estimated using a data association technique with the TOA results. Similarly, [21] estimates the possible location of the target, and performs tracking the target’s path using an association based on time. These methods perform an association to estimate the locations where the actual targets exist, there is a possibility of estimating a position that is completely different from the position, in which the actual targets exist, depending on the distance values of targets detected in the radar or the environment of the radar coverage.

In this study, a radar system is designed to detect and track multiple targets behind the walls by penetrating the composite material wall made of bricks and concrete. It is difficult for delay-and-sum, which is a commonly used algorithm in through-wall applications, to estimate and track the targets’ locations, as the image result of this algorithm varies rapidly according to the state of targets and surrounding environment of radar coverage. Accordingly, the objective of this study is to propose a novel method, which estimates the location of multiple targets by overcoming the aforementioned drawbacks. This method performs the detection by using the constant false alarm rate (CFAR) algorithm on several radars and calculates the probability of target existence at a certain location using the likelihood.

This paper is organized as follows: In the second section, the entire algorithm processes and hardware specifications of the through-wall radar system are presented. Section 3 is about the proposed algorithm for position estimation and performance comparison through the simulation results for different scenarios. The experimental results are shown in Section 4 and we conclude the paper in Section 5.

## 2. Through-Wall Radar System

The through-wall radar system is designed to detect targets that exist behind the wall as well as estimate and track their position. In this section, the hardware configuration and overall algorithm of the through-wall radar system are briefly explained.

### 2.1. Hardware Design

**Figure 1:**
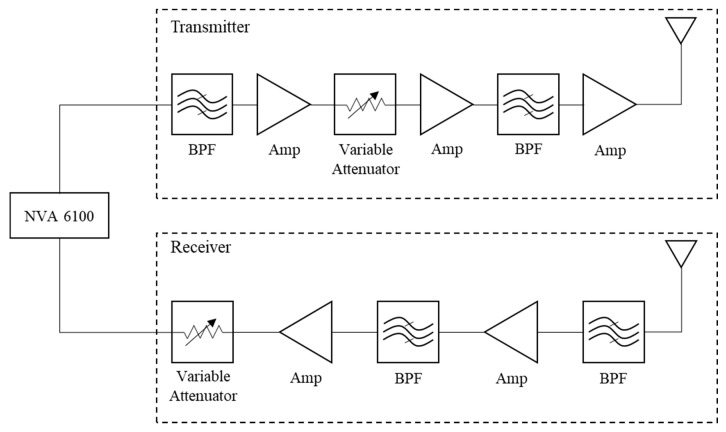
Ultra-wide band radar module.

Our through-wall radar system uses a NVA6100 single chip impulse-based radar transceiver manufactured by NOVELDA (Novelda AS, Kviteseid, Norway). Table 1 shows NVA6100 specifications the NVA6100 chip operates in the range of 0.4–3.2 GHz by exploiting a low-band Gaussian pulse generator. As observed from Figure 1, the generated Gaussian pulse passes through filters and amplifiers to obtain the appropriate amount of power to detect targets behind the wall. The effective isotropic radiated power of the entire system is 46 dBm with a noise figure of 6 dB. We can represent 17 dBm as the average power in our radar system for military and specific applications like search and rescue. These radar system specifications are designed to suit the characteristics of the wall, which was presented as a brick-faced concrete wall in [22].

**Figure 2:**
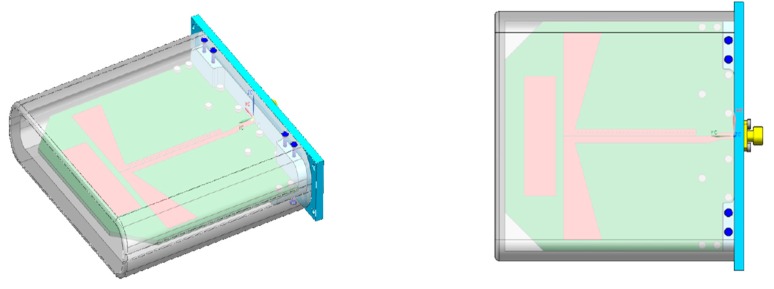
Half bow-tie quasi-Yagi antenna.

The antennas used in our radar system are the half bow-tie quasi-Yagi type, which are designed to handle a relatively wide coverage area. If an antenna with narrow coverage is applied, it demonstrates a relatively high peak power. However, the joint coverage of overall antennas is narrow. The narrow joint coverage reduces the area, in which the radar system detects the targets or detection performance. Therefore, we applied wide beam antennas as represented in Figure 2 to maximize the coverage of the through-wall radar system.

The complete radar system comprises four IR-UWB radar modules and antennas. It was designed to be installed in front of the wall for conducting the detection and tracking behind the walls.

### 2.2. Through-Wall Radar System Process

**Figure 3:**
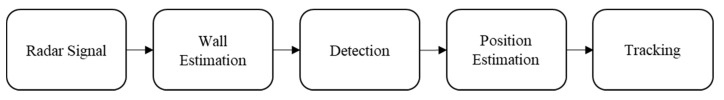
Radar system block diagram.

In our radar system, the radar signal received from the four radar modules (as described earlier) was processed sequentially to determine the situation behind the wall. As the main scope of this study involves proposing a novel positioning method that can be applied to through-wall applications, we describe the operation of each algorithm block briefly in this section. The block diagram of the radar system is shown in Figure 3.

The first process preformed in our through-wall radar system was the wall estimation, which estimated the characteristic of the wall to compensate for the effects of the wall. Generally, it is not easy to recognize the situation behind the wall, as the radar signal experiences reflection, refraction or diffraction, when the signal passes through the wall [23]. The effect of clutter influences the radar signal, which passes through the wall, and the distortion of the received signal increases due to the multi-reflection within the wall. Furthermore, the amplitude of the signal reflected by the targets decreases in comparison to the its radar cross section due to the occurrence of absorption.

In addition to distortion and attenuation, the distance of the target detected by the radar may also contain errors, as the radar signal travels more slowly than propagates in the air. This distance error can be represented using the thickness and dielectric of the wall as mentioned in [24]:

(1) $$\mathrm{TOA}\left(d\right)=\frac{d_{A}}{c}+\frac{d_{W}}{v_{W}},$$

(2) $$v_{W}=\frac{c}{\sqrt{\varepsilon_{W}}},$$

where the total distance d includes the distance in the air $d_{A}$ and distance through the wall $d_{W}$. The speed of light and velocity of radar signal penetrating the wall are represented as c and $v_{W}$, respectively. In addition, $v_{W}$ can be deduced using the speed of light and dielectric of the wall. The distance measured by the radar increases with $d_{W}$ and $\varepsilon_{W}$, which are the thickness and dielectric of the wall, respectively.

In the wall estimation step, the radar system extracted the characteristic of received signal and then estimated the existence of obstacles along with the dielectric and thickness of the wall using machine learning. As previously mentioned, the signal emitted from radar lowered its speed while penetrating the wall, and therefore, the distance measured by the radar could contain errors. Under these circumstances, if the dielectric and thickness of the wall were known, then the actual distance of the target could be estimated by compensating for them. In our through-wall radar system, the dielectric was estimated with the envelope of the received radar signal using support vector machine. The thickness of the wall was obtained with both the envelope and frequency components of the received radar signal by exploiting the neural network.

The second step included the detection process, which detected the targets from the received radar signal. The received signal form can be represented as mentioned in [25]:

(3) $$r_{k}\left(t\right)=\overset{N}{\underset{i=1}{\sum }}a_{ki}s\left(t-\tau_{ki}\right)+n\left(t\right),$$

where s(t) and n(t) denote the transmitted signal and noise of the channel, respectively. The subscript k denotes the slow time index, which represents the k-th received signal. The subscript t denotes the fast time index, which represents the time utilized to receive the transmitted signal. The radar signals are attenuated by $a_{ki}$ and delayed by $\tau_{ki}$, while they travel through N paths due to multipath effect.

**Figure 4:**
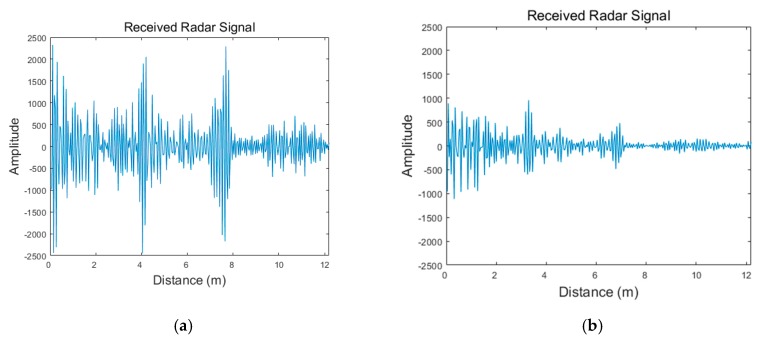
Radar signals at detection stage: (**a**) received radar signal with no wall; (**b**) received radar signal through the wall.

Figure 4 presents the signals received from the radar with and without the presence of a wall. As observed from this figure, the two signals show entirely different aspects. The signal received through the wall shows that the overall signal amplitude is significantly attenuated compared to the signal received without the wall.

**Figure 5:**
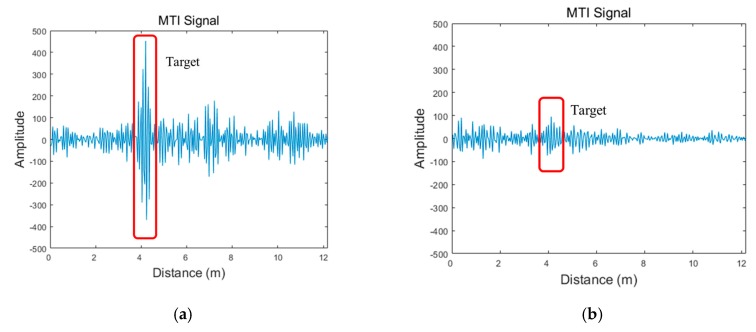
Radar signals at detection stage: (**a**) moving target indicator (MTI) signal with no wall; (**b**) MTI signal through the wall.

A human target was placed approximately 4 m away from the radar. However, it was difficult to recognize the target from the received radar signal. In these situations, a special filter, called the moving target indicator (MTI) is generally used for extracting the target signal from the received radar signal. The recursive filter, one of the MTI algorithms used for this purpose, generated the clutter-suppressed signal by subtracting the estimated clutter signal from the received radar signal as suggested in [26] such that:

(4) $$c_{k}\left(t\right)=y_{k-1}\left(t\right)+c_{k-1}\left(t\right),$$

(5) $$y_{k}\left(t\right)=r_{k}\left(t\right)-\left(1-\alpha \right)c_{k}\left(t\right),$$

(6) $$=r_{k}\left(t\right)-\left(1-\alpha \right)\overset{k-1}{\underset{n=1}{\sum }}\alpha^{n-1}r_{k-n}\left(t\right),$$

where $c_{k}\left(t\right)$ represents the estimated clutter signal at the k-th scan updated recursively with clutter eliminated signal at k−1-th $y_{k-1}\left(t\right)$ and the previous estimated clutter signal $c_{k-1}\left(t\right)$. Although the estimated clutter signal also contains signal from targets to be detected, most of components are composed of clutter, which is determined based on the application ratio α. If the application ratio is set to be high, the target signal has relatively high amplitude in the clutter eliminated signal but requires more time to obtain the estimated clutter signal which reflects the circumstance of radar coverage properly. The results of these two signals processed with MTI are shown in Figure 5. In this figure, we clearly observe the presence of the target, when the wall does not exist. However, the target signal is significantly attenuated, rendering it difficult to identify it from the MTI signal due to the effects caused by the wall and clutter in the through-wall case.

After extracting the target signal, the targets should be detected from the MTI signal. If the radar coverage was clear, or the background was empty, then the detection process could simply operate with values over a certain noise power. However, in practice, phenomena, such as ghost, may occur due to the environmental changes caused by the movement of targets or clutter. In particular, it is not easy to recognize the target signal due to wall-included reflection and signal attenuation in the through-wall radar system. CFAR is the most commonly used algorithm in radar systems for detection of the target. The CFAR algorithm performs target detection within a certain false alarm rate by exploiting the statistical characteristics of the radar signal [27].

**Figure 6:**
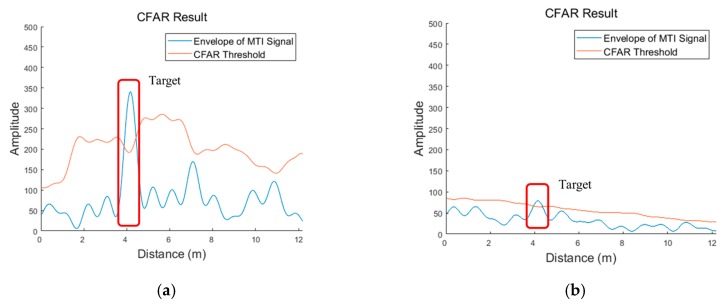
Radar signals at the detection stage: (**a**) constant false alarm rate (CFAR) result with no wall; (**b**) CFAR result through the wall.

Figure 6 shows the results of the CFAR algorithm. The CFAR threshold is generated by estimating the statistical characteristics of the clutter and noise of the radar signal. The target is estimated to exist in the area, where the radar signal is higher than the CFAR threshold. As observed from this figure, the target signal is relatively higher than the CFAR threshold in the radar signal, when there exists no wall. However, there is only a marginal difference between the target signal and the threshold in the radar signal received through the wall. This implies that it is difficult to distinguish between the target signal and signal caused by the effect of the wall and clutter. Detecting targets through the wall demonstrates a lower detection rate and false alarm rate than in general cases. Therefore, it is difficult to apply the traditional and commonly used positioning techniques to achieve the required results. However, our proposed algorithm is designed to operate appropriately with a relatively unstable detection. The location of the targets was estimated in the position estimation step by exploiting the information on the targets detected through the detection process.

Generally, the trilateration (solved with the least squares) method is the most commonly used technique to estimate the location of a target using distance values [28]; however, it is not easy to estimate the locations of multiple targets using the radar with this method. The trilateration method can be applied, only when the position of radars and distance values of the target detected on each radar are known. However, it is difficult to recognize which distance value belong to a certain target, when the radar detects multiple targets. There have been many attempts to estimate the location of multiple targets, we discuss about these methods in Section 3.

In the last step of tracking, the moving path of the estimated target was expressed as a track to track a certain target. This was performed to overcome the problem related to the estimated target positions still encountering missed detections and false alarms and track the location of the targets more precisely. To track targets, the track was generated by exploiting the location of the estimated targets obtained from the previous step of position estimation. This step included the track initiation and track management processes.

The track initiation process indicated the area, in which the target would exist the next time, as the gate area using the prediction obtained according to the previous location and movement of the target. If the target was detected in this gate area, the track was generated by linking it to the location of the target obtained previously. These generated tracks were determined values that belong to a certain time window. It was assumed that the actual target existed, where the threshold exceeded. In the track management step, track termination was performed, if there was no update of track, because the target had not been detected for a certain period of time, or the track possessed a value under a certain threshold [29]. The determination of tracks for the actual target through track initiation and track management processes were performed using a Kalman filter [30].

**Figure 7:**
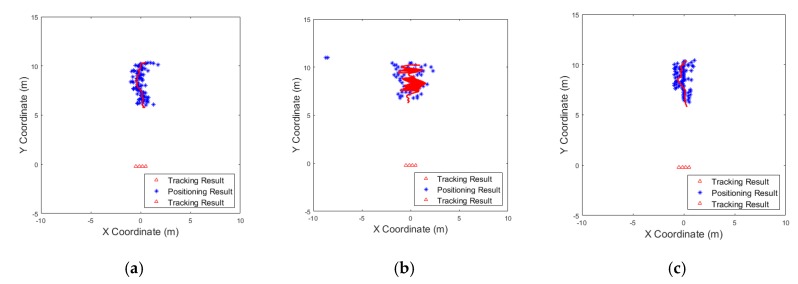
Example of tracking performances for different positioning methods: (**a**) trilateration; (**b**) delay-and-sum; (**c**) proposed algorithm.

Figure 7 shows tracking results for different positioning methods performed with the experiment when one target moves back and forth. In all three results, the same tracking approach was applied. The blue dots in the figure denote the position estimation results within a certain time period, and the red line denotes the results of Kalman tracking, which is determined as the actual track. As observed, the moving path of the target can be tracked accurately through the tracking process. Tracking stage was the last process in our radar system, and therefore, the final results were generated in this stage.

## 3. Positioning Algorithm

In this section, the methods for estimating targets’ position named as trilateration, delay-and-sum and the method proposed in this study, are introduced and compared.

### 3.1. Trilateration Algorithm

As mentioned in Section 2, it is not easy to combine distance values for the same target in a multiple target environment. To solve this problem, there have been many attempts to estimate the location of multiple targets using distance values. In [31], all possible locations are discovered by the combination of distance values using the least squares method, regardless of which target the detected distance value denotes. For all these estimated possible locations, a specific method named as association is used to estimate the positions of the actual targets using the difference between the distance to each radar and detected distance value. In contrast, a slightly different method is used in [21], in which a combination of the distance values is used (similar to the one proposed before) to discover possible locations, and the locations with smooth path are estimated as actual targets. Estimating the locations of multiple targets using these methods is very effective; however, when the distance value or the number of detected targets in each radar is different due to miss detection or false alarm in detection stage, these methods should generate the combination of distance value to be used to estimate all possible positions, which is very complex and has many considerations. If there is in an error within these processes, there is a possibility of estimating positions which are completely different from the actual positions of the targets. Therefore, these approaches are not easy to apply in through-wall applications, as it is difficult to reliably detect targets and not suffer from false alarms due to dense clutter in these environments.

Another algorithm in [20] shows a different aspect, which uses radar deployment conditions to perform association. This method combines only the distance values within a certain distance determined by the radar deployment from the distance value of a target. This uncomplicated association method is highly efficient and demonstrates good performance, however, there is still a possibility that it cannot perform properly when the distance between the targets is too close. Since this method is more suitable for our through-wall radar system, where the distance between radars is close, we apply this method for trilateration when comparing the performance between positioning algorithms.

### 3.2. Delay-and-Sum Algorithm

The through-wall radar applications generally operate by generating the radar images using the delay-and-sum algorithm. This algorithm functions by adding the target signal extracted through the MTI algorithm to the radar coverage area [32]:

(7) $$I_{x,y}=\overset{N}{\underset{i=1}{\sum }}y_{i}\left(d_{x,y}\right).$$

The image result at the Cartesian space (x, y) is represented as $I_{x,y}$, and $y_{i}\left(d\right)$ denotes the value at the distance d of MTI signal on the ith radar. The distance from the radar to (x, y) is represented as $d_{x,y}$, and therefore, $I_{x,y}$ is the cumulative form of the MTI signal from each radar.

As the delay-and-sum method uses the MTI signal as is, the result image is not stable over time, as it is sensitive to changes in the radar cross section depending on the target state or movement. Furthermore, if the target’s movement is minimal or the attenuation effect is high due to the wall, the signal produced by the clutter may be greater than that of the target. Therefore, it is not easy to identify the target in the image result. Therefore, extracting the coordinate of targets from the image using the delay-and-sum method suffers the risks of poor detection rate and false alarm rate.

### 3.3. Proposed Algorithm

To overcome the limitations of the conventional positioning method and detect targets with improved detection rate and false alarm rate, we proposed a technique to estimate the targets’ location through the detection process in radar using the likelihood that the targets exist at a certain distance.

**Figure 8:**
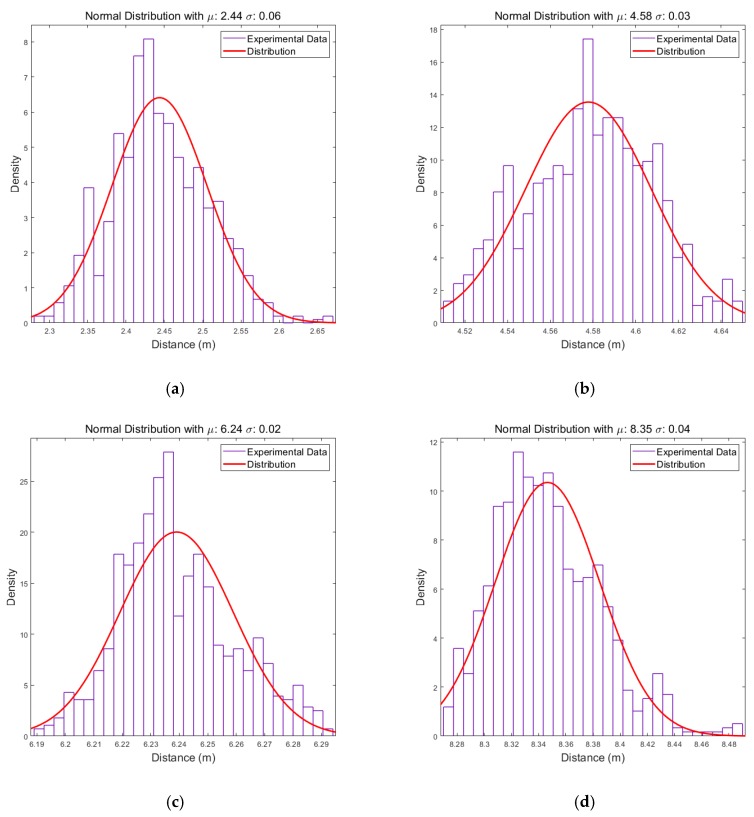
Distribution of distance of the detected target: (**a**) the target is placed approximately 2 m away from the radar; (**b**) the target is placed approximately 4 m away from the radar; (**c**) the target is placed approximately 6 m away from the radar; (**d**) the target is placed approximately 8 m away from the radar.

When a target is detected through the CFAR algorithm, the distance value of the target can be extracted, which does not possess a fixed value and varies over time. Figure 8 shows the histograms of the distance values of detected target through the wall corresponding to time and an approximation of their distribution.

Each approximated distribution is in the form of a normal distribution and exhibits a standard deviation of approximately 0.03, except when the target is very close. In addition, the expectation values do not represent the exact distance, which is due to the system setup and wall effect. However, they do not affect the distribution characteristics exploited by the proposed algorithm. Using these approximated distributions, when a target is detected at a certain distance, the probability of the target existence according to the distance can be expressed as a likelihood. The cumulative likelihood can also be generated by accumulating the probability that the targets detected from each radar to the radar coverage, and this can be used to estimate the targets’ location with multiple radars.

The proposed Algorithm 1 used the CFAR algorithm in the detection stage to exploit the probability that the existence of the target is based on the distance from the grids generated in the initialization process to each radar in the form of the recursive cumulative likelihood.
**Algorithm 1 Position Estimation with the Proposed Algorithm**1. Initialization Generate grids ${G_{x,y}^{t}|}_{t=0}$ for x=1, …, X and y=1, …, Y, considering the coverage and performance of the radar system. For example, in our radar system, one grid is a square of 0.2${\;m}^{2}$.
2. For x=1, …, X and y=1, …, Y, calculate the cumulative likelihood as follows:
(8)$$G_{x,y}^{t}=G_{x,y}^{t-1}\overset{N}{\underset{i=1}{\sum }}\overset{M}{\underset{k=1}{\sum }}ℒ(p_{x,y}|z_{i,k})\;,$$
$ℒ(p_{x,y}|z_{i,k})$ is the likelihood function of a particular point $p_{x,y}$ in grids, when the kth target is detected by the ith radar. $G_{x,y}^{t}$, which is the grid value at time t, is generated by recursively exploiting $G_{x,y}^{t-1}$. A probability of target existence of a particular point can be shown through these generated grids $G_{x,y}^{t}$.
3. For x=1, …, X and y=1, …, Y, normalize the cumulative likelihood for recursive operation as follows:
(9)$$G_{x,y}^{t}=\frac{G_{x,y}^{t}}{\sum_{x=1}^{X}\sum_{y=1}^{Y}G_{x,y}^{t}},$$
The normalizing process is required to make the sum of grids is to be one, and determine the effectivity of grids regarding re-initialization.
4. Find the coordinates over the threshold to estimate the location of multiple targets such that
$$G_{x,y}^{t}>G_{thresh}\;,$$
$G_{thresh}$ is set adaptively according to the average number of targets detected by the radars. This is because if there is a large number of targets detected by the radars, the value of normalized probability of target existence represented by the cumulative likelihood will be reduced.
5. Calculate the effectivity of grids as follows: (10)$N_{eff}=\frac{1}{\sum_{x=1}^{X}\sum_{y=1}^{Y}{\left(G_{x,y}^{t}\right)}^{2}}\;,$

The effectivity of grids represents that how the values in grid are distributed, if the cumulative likelihood converges on one particular grid, the other grids possess values close to zero. Therefore rendering the recursive operation using the grids of the previous time difficult to operate appropriately. In such a case, the value of $N_{eff}$ declines. Re-initialization process will be performed when $N_{eff}<N_{thresh}$, as the recursive process is deemed difficult to operate properly.

### 3.4. Performance Comparison by Simulation

We performed simulations according to the various scenarios for comparing the performance of three positioning algorithms. We deployed the radars very close, and generated radar signals with Gaussian pulses reflected on the target and received with a certain noise. The target signal was extracted through MTI algorithm, and the detection process was performed with a detection rate of 75% and false alarm rate of 10% in each radar similar to the actual experimental environment. Also, the distance error of detected targets was followed by the normal distribution with a standard deviation of 0.03 m as seen in our radar system. Overall parameters related to the simulation are summarized in Table 2.

The simulation scenarios are summarized in Table 3. There are various scenarios for multiple targets and for moving targets, including the first scenario as a reference for evaluating the performance of the algorithms. Scenarios for multiple targets are designed to assess whether each algorithm is able to distinguish the targets, and scenarios for moving targets are designed to evaluate whether each target can be properly tracked as it moves. The results are presented as the output of the entire radar system algorithm as described in Section 2.

The results are expressed in terms of detection rate, false alarm rate and positioning error for the target according to each scenario. The detection rate $P_{d}$ refers to the number of targets that are correctly detected corresponding to the number of targets that actually exist, and the false alarm rate $P_{f}$ refers to the number of false alarms occurring for opportunities of error [33]. The positioning error denotes the difference between the position, where the target actually exists, and the estimated position of target, which is expressed through mean squared error. In our results, if the location of the estimated target is within the radar coverage, it is determined to be detected, and if the location of the estimated target is outside the radar coverage or the number of detected targets is greater than the actual number of targets, it is determined as false alarm. Positioning error is calculated exploiting the distance difference between the estimated position determined to be detected and the actual location of targets:

(11) $$P_{d}=\frac{\mathrm{number}\;\mathrm{of}\;\mathrm{correct}\;\mathrm{target}\;\mathrm{detections}}{\mathrm{number}\;\mathrm{of}\;\mathrm{actual}\;\mathrm{targets}},$$

(12) $$P_{f}=\frac{\mathrm{number}\;\mathrm{of}\;\mathrm{flase}\;\mathrm{alarms}}{\mathrm{number}\;\mathrm{of}\;\mathrm{false}\;\mathrm{alarm}\;\mathrm{opportunities}},$$

**Figure 9:**
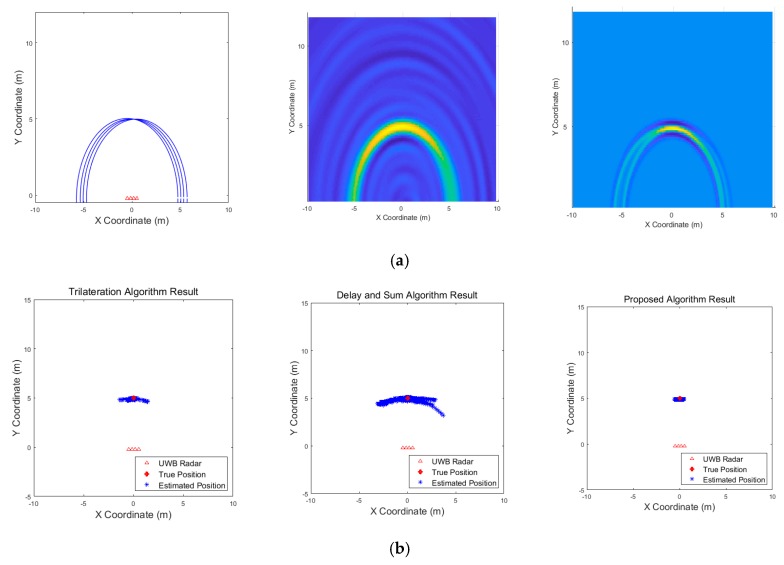
Simulation results when one target stands (Scenario 1): (**a**) instantaneous results of position estimation step according to the positioning algorithm; (**b**) results of the radar system for the entire simulation.

**Figure 10:**
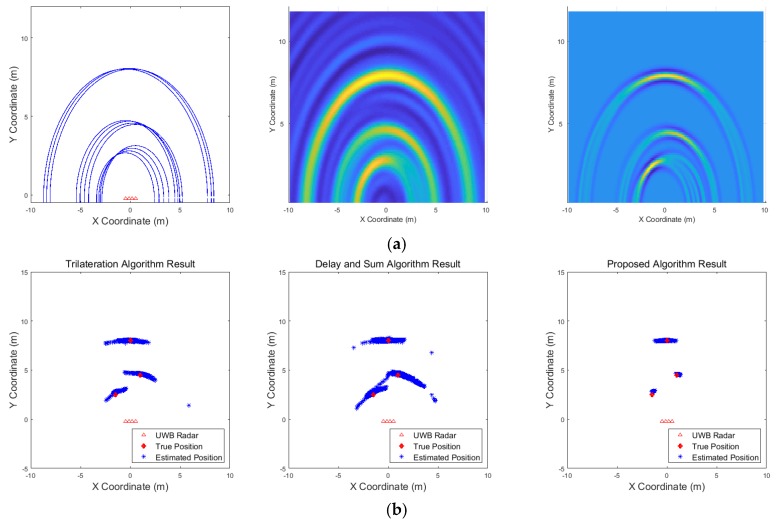
Simulation results when three targets stand (Scenario 2): (**a**) instantaneous results of position estimation step according to the positioning algorithm; (**b**) results of the radar system for the entire simulation.

**Figure 11:**
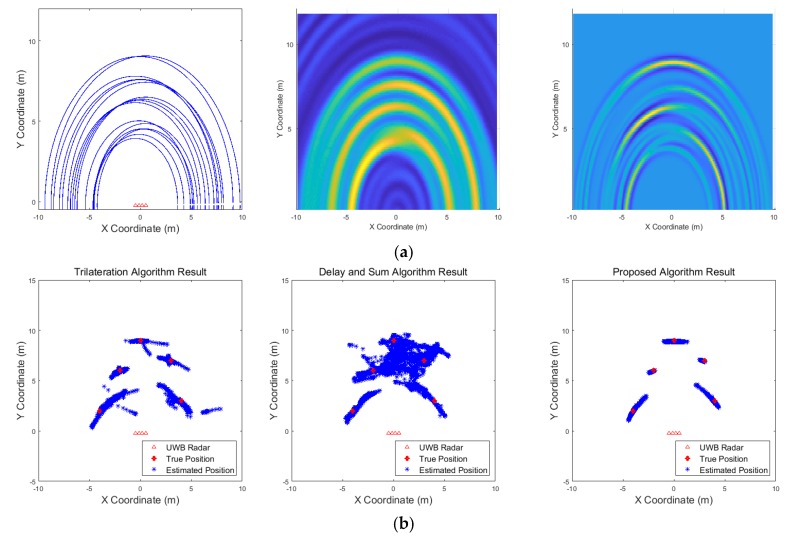
Simulation results when five targets stand (Scenario 3): (**a**) instantaneous results of position estimation step according to the positioning algorithm; (**b**) results of the radar system for the entire simulation.

**Figure 12:**
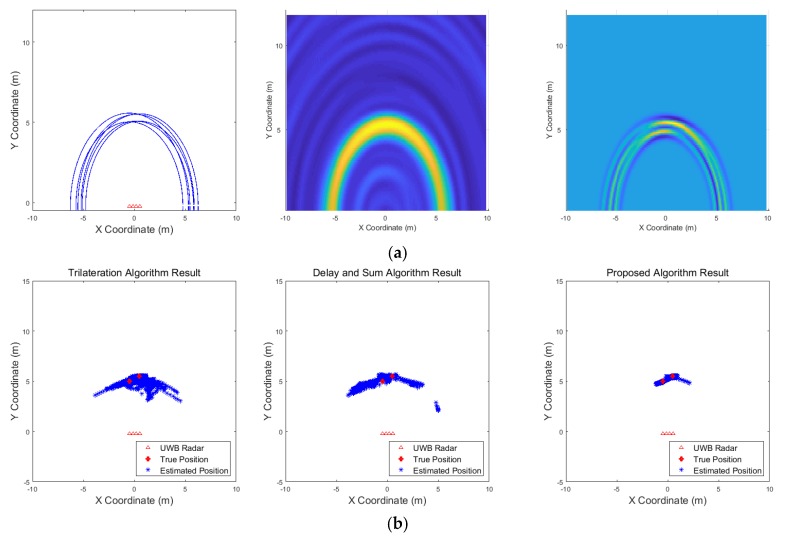
Simulation results when two targets stand close each other (Scenario 4): (**a**) instantaneous results of position estimation step according to the positioning algorithm; (**b**) results of the radar system for the entire simulation.

**Figure 13:**
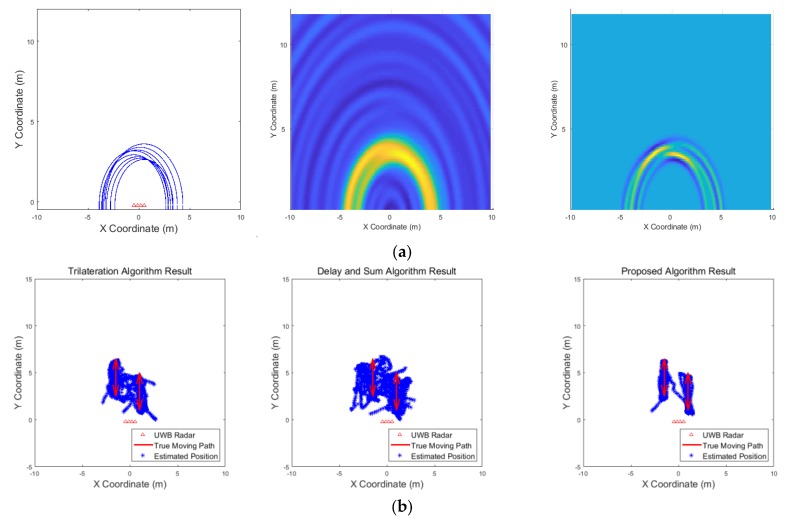
Simulation results when two targets move (Scenario 5): (**a**) instantaneous results of position estimation step according to the positioning algorithm; (**b**) results of the radar system for the entire simulation.

**Figure 14:**
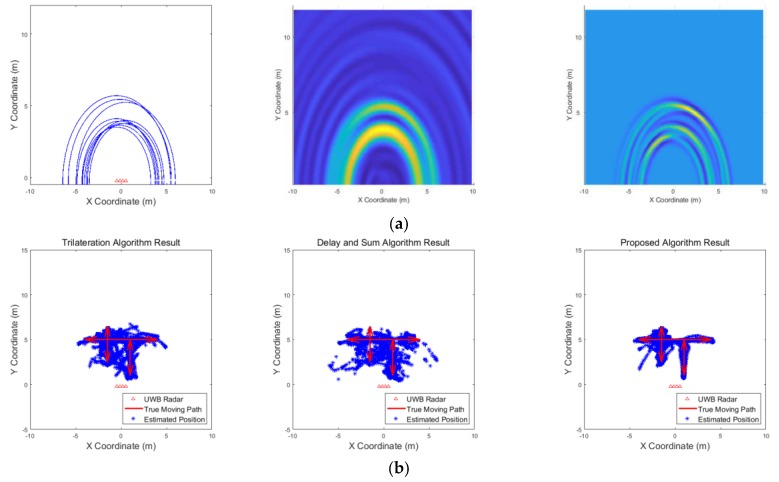
Simulation results when three targets move (Scenario 6): (**a**) instantaneous results of position estimation step according to the positioning algorithm; (**b**) results of the radar system for the entire simulation.

**Figure 15:**
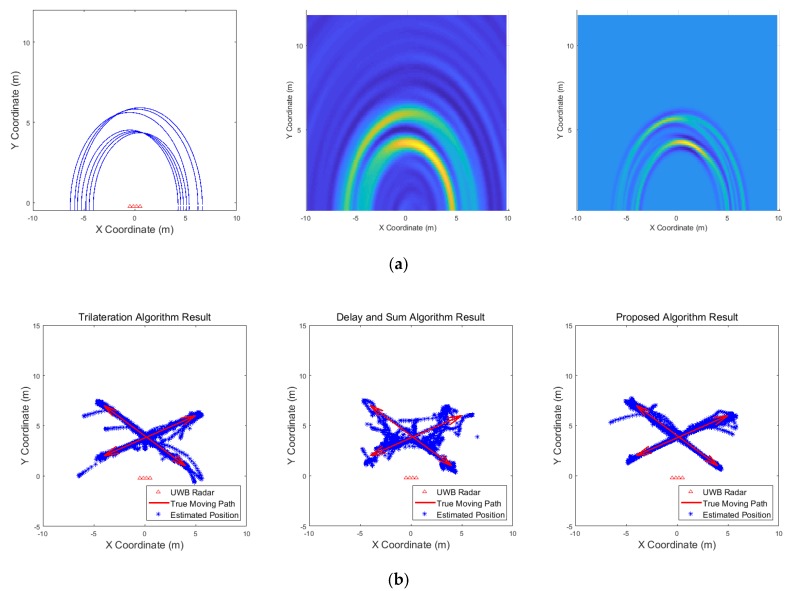
Simulation results when two targets move cross over (Scenario 7): (**a**) instantaneous results of position estimation step according to the positioning algorithm; (**b**) results of the radar system for the entire simulation.

**Figure 16:**
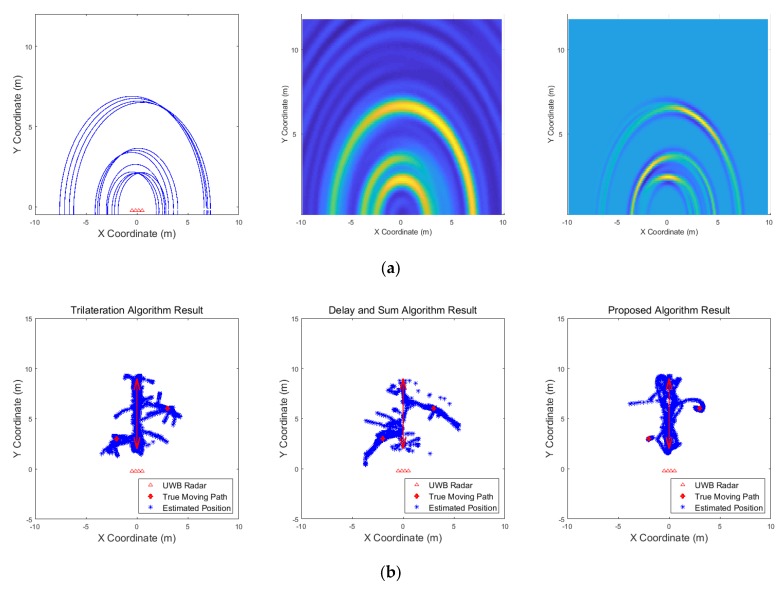
Simulation results when two targets move and one target stands (Scenario 8): (**a**) instantaneous results of position estimation step according to the positioning algorithm; (**b**) results of the radar system for the entire simulation.

**Figure 17:**
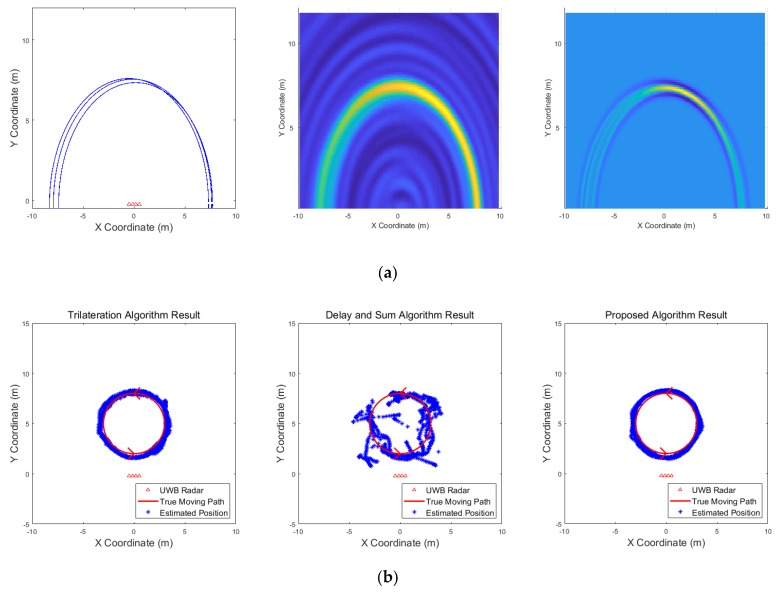
Simulation results when one target moves in a circle (Scenario 9): (**a**) instantaneous results of position estimation step according to the positioning algorithm; (**b**) results of the radar system for the entire simulation.

**Figure 18:**
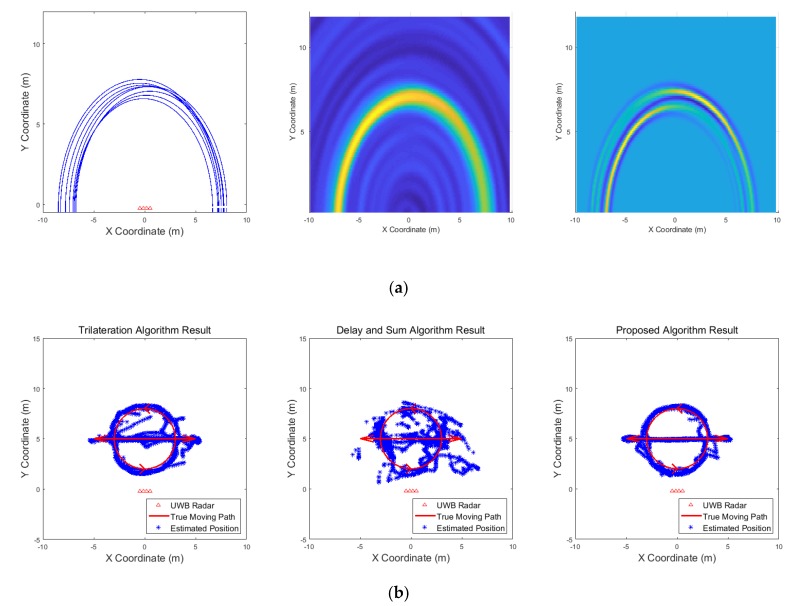
Simulation results when one target moves in a circle and one target moves horizontally (Scenario 10): (**a**) instantaneous results of position estimation step according to the positioning algorithm; (**b**) results of the radar system for the entire simulation.

Figure 9, Figure 10, Figure 11, Figure 12, Figure 13, Figure 14, Figure 15, Figure 16, Figure 17 and Figure 18 show the results according to each scenario, and the numerical results are summarized in Table 4. The instantaneous results in each figure represent the results of position estimation step according to the positioning algorithm. The delay-and-sum algorithm and the proposed algorithm generate the image and the cumulative likelihood, respectively. The result of the delay-and-sum algorithm shows that the image is generated for the entire area of radar coverage and interfered by noise signal. In contrast, in case of the proposed algorithm, the values of the grids were calculated only in the area, where a target was detected. Moreover, the use of likelihood, unlike the radar signal, was not affected by the target’s radar cross section, resulting in a clear form.

In comparison to the scenario in Figure 9, the scenarios in Figure 10 and Figure 11 dealing with multiple targets generally show lower detection rates, higher false alarm rates and position errors. Compared to the poor performance of the delay-and-sum algorithm as the number of target increases, the trilateration and proposed algorithm show relatively good performance. In particular, the delay-and-sum method in Figure 11 shows high detection rate, however, it also has very high false alarm rate and position error, which makes it difficult to say that this method shows good overall performance. However, as the number of targets increases, positioning error of the trilateration results increases dramatically. In Scenario 4, where the distance between targets is very close as shown in Figure 12, it can be seen that the association method which combines the distance values within a certain distance used in the trilateration algorithm, is difficult to operate correctly, resulting in high false alarm rate. The trilateration and proposed algorithm show good performance in the scenarios from Figure 13, Figure 14 and Figure 15 where the targets are moving, while the delay-and-sum method shows low detection rate or high false alarm rate with relatively high position error. In Scenario 8, similar to Scenario 4, the trilateration algorithm shows high false alarm rate as the association process is not easy to operate properly according to the movement of the target as represented in Figure 16. In addition, when a small number of targets are moving, it can be reaffirmed as a result of Scenario 9 and 10 that the trilateration and proposed methods perform well as shown in Figure 17 and Figure 18.

To sum up, the trilateration algorithm shows very good performance when the number of targets is small, however, this method has difficulty in situations that does not satisfy the conditions of applied association method. The delay-and-sum algorithm uses the radar signal as is, the image varies rapidly depending on the condition of the target. Since it is difficult to predict the result of the image, therefore high detection rate and low false alarm rate is not easy to be obtained at same time. In contrast, the proposed method is not affected by changes in the radar signal, unlike the delay-and-sum algorithm, since only the detected targets are calculated. It also does not need an association process, so it is free from errors that occur in this process. We can see that the proposed algorithm shows good performance in the simulations we have performed, especially in the multiple target environments.

**Figure 19:**
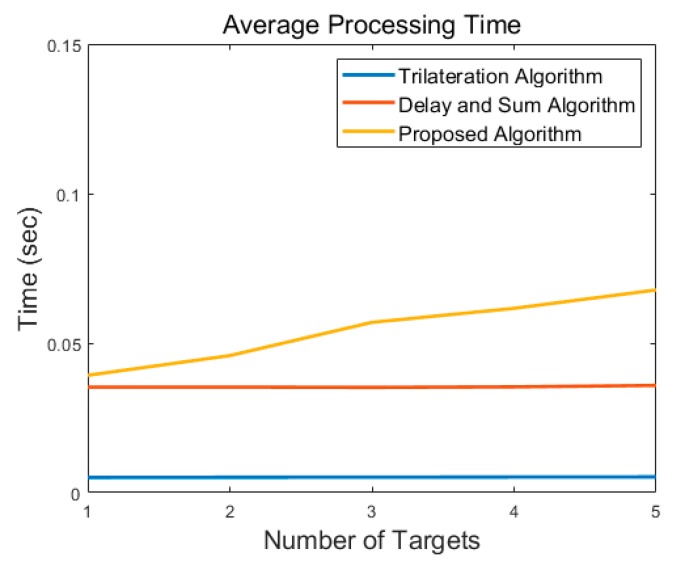
Comparison of processing time according to number of targets.

Figure 19 represents average processing time of each algorithm according to number of targets. The trilateration algorithm with the association method introduced in [20] shows the minimum processing time, as this method only operates with distance values after detection step using CFAR. The delay-and-sum algorithm shows the relatively high processing time, due to exploiting the MTI signal as is. The proposed algorithm requires more processing time, as it performs detection process using CFAR and then calculates the likelihood for the detected target. However, the actual computational time required by the proposed algorithm does not account for much of the overall system operation, it is possible to configure the real-time through-wall radar system.

## 4. Experimental Results Based on the Designed Through-wall Radar Hardware

**Figure 20:**
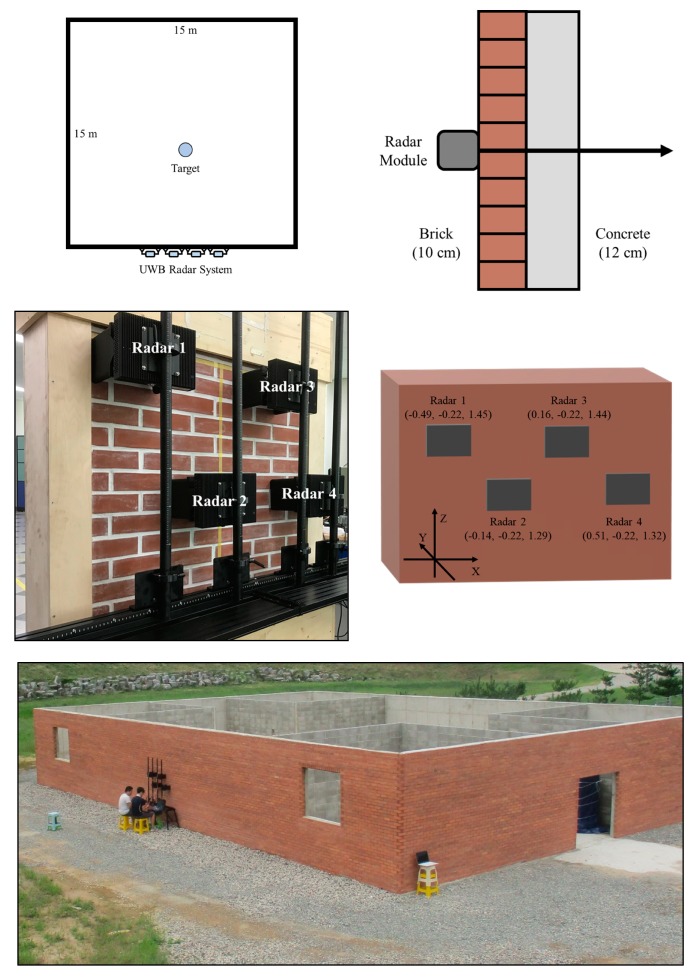
Experimental setup.

After designing the through-wall radar system, we conducted experiments at the Gimcheon test site to test the performance of this system. As shown in Figure 20, the targets are placed in a square-shaped building surrounded by walls that are 15 m long to address several scenarios, and our radar system is installed in front of the outer wall of the building. The wall of the building is made of a composite material comprising approximately 10-cm-thick bricks and 12-cm-thick concrete. The total thickness of the wall is 22 cm similar to the brick-faced concrete wall used in [22].

The experimental scenarios conducted are listed in Table 5. These scenarios are designed to assess the performance of the radar system under situations, such as when the number of targets increases and the target is moving and when one target is placed. Each scenario lasted for approximately 5 min, and the results are presented as the output of the entire radar system algorithm, as described in Section 2. In addition, to show the validity of the position estimation algorithm proposed in this study, the results using the trilateration algorithm in [20] and the commonly used delay-and-sum algorithm are also obtained and compared.

**Figure 21:**
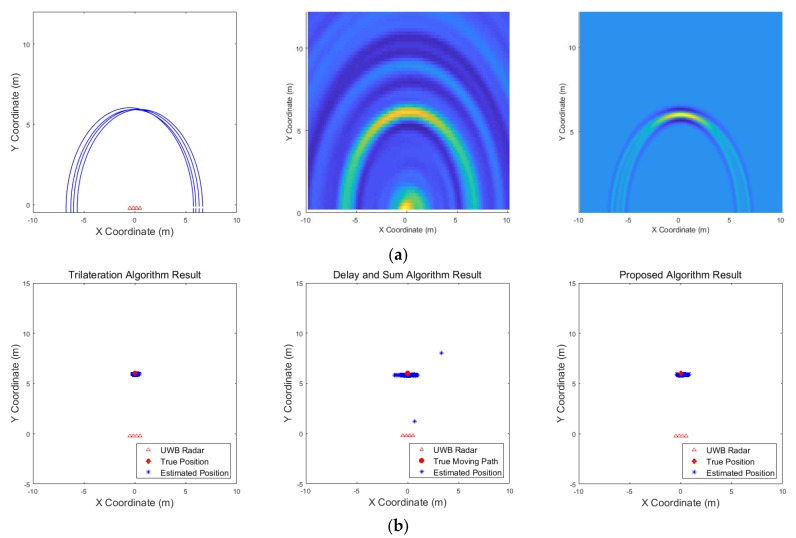
Experimental results when one target stands 6 m from the wall (Scenario 1): (**a**) instantaneous results of position estimation step according to the positioning algorithm; (**b**) results of the through-wall radar system.

**Figure 22:**
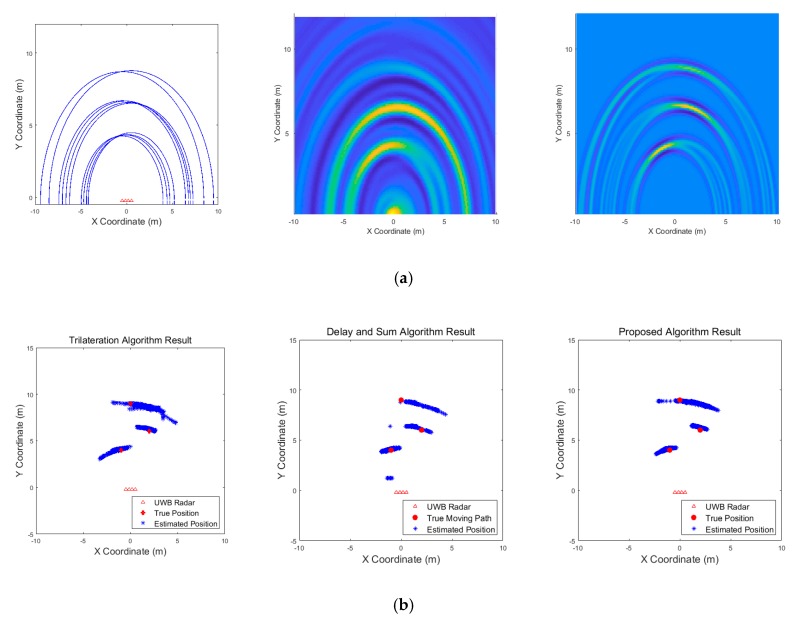
Experimental results when three targets stand behind the wall (Scenario 2): (**a**) instantaneous results of position estimation step according to the positioning algorithm; (**b**) results of the through-wall radar system.

**Figure 23:**
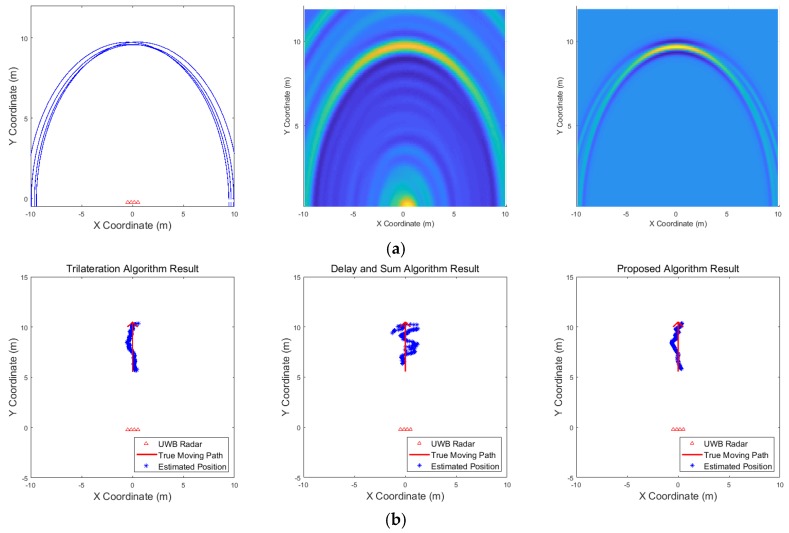
Experimental results when one target moves back and forth behind the wall (Scenario 3): (**a**) instantaneous results of position estimation step according to the positioning algorithm; (**b**) results of the through-wall radar system

Figure 21 shows the results corresponding to the three algorithms for the scenario of one target standing. The result of the delay-and-sum algorithm shows that there are false alarms due to multipath effect at the back of location, where the target actually exists. There are other false alarms due to the wall, and the estimated positions appear in a relatively large area. In contrast, according to the result obtained from the proposed algorithm, there are no false alarms due to the wall or multipath effect, and the estimated positions are closer to the actual target. The trilateration algorithm also shows good result in this scenario. Figure 22 shows the results for the scenario of three targets standing. In this case, the results obtained from the delay-and-sum algorithm suffers false alarms due to the wall and multipath effect. The trilateration algorithm and the proposed algorithm show similar aspect in this case, however, the estimated positions obtained by the trilateration algorithm appear in a large area. Finally, the results of a single target moving back and forth are shown in Figure 23. This figure indicates only one path from forward to backward to clearly identify the path of the target’s movement. In this case, the drawback of the delay-and-sum algorithm is highlighted, as the target’s movement causes a rapid variation in the target’s radar cross section. Therefore, the image produced as a result of this algorithm also changes rapidly, rendering the path of the target highly inaccurate. However, the results obtained using the trilateration and the proposed algorithm show a more stable path without being significantly affected by the variation in the target’s radar cross section.

The numerical results of these three scenarios are listed in Table 6. In scenario 1, when one target stands, all three algorithms function appropriately with the trilateration algorithm and the proposed algorithm showing marginally better performance than the delay-and-sum algorithm. In other scenarios, a slightly different phenomenon can be observed. Severe multipath effect increases the number of false alarms and decreases the detection rate of targets in the second scenario. The delay-and-sum method shows poor performance due to wall and multipath effect, and the trilateration algorithm also shows rapid change in performance as the number of targets increases. This is due to miss detection or false alarm occurring in the detection process of each radar, and this leads to the estimation of positions that are different from where the actual targets exist, resulting in high false alarm rate in the numerical result. While the trilateration and proposed algorithm show relatively good performance in the last scenario in which the target moves, the delay-and-sum algorithm suffers from false alarms due to rapid changes in radar signals. In addition, the first scenario of simulations and experiments are performed for the same position of the target, however, show slightly different results. The trilateration method shows the best performance in both results, this aspect becomes more apparent in the experimental results. This is due to different aspects of miss detections and false alarms. While the simulations show results for the more general environment, the results of the experiments are greatly affected by the environmental characteristics of the radar system. In the first scenario of the experiments, the number of miss detections and false alarms occurring around the target on each radar is relatively small due to the environmental characteristics. Therefore, the detection rate, false alarm rate and position error of the numerical results are better than the simulation results. In contrast, in the second scenario of the experiments, the environmental characteristics of the radar system, including the clutter, and the multipath effect result in frequent miss detections and false alarms, the results are much worse than the simulations. In particular, as shown in Figure 22, the trilateration method is greatly affected by these miss detections and false alarms occurring around the target. To sum up, it is established that the proposed algorithm performs better than the delay-and-sum algorithm, as it is less affected by the clutter due to the state of the radar coverage. The trilateration algorithm shows the best performance in the single target scenario, however, as shown in the simulation results in Section 3, this algorithm shows relative lower performance in the multiple target scenario. Consequently, the location of the multiple targets can be estimated more accurately using the proposed algorithm, while maintaining a relatively high detection rate and low false alarm rate. 

## 5. Conclusions

In this study, we designed the through-wall radar system and conducted experiments to evaluate its performance. A novel technique was also proposed to improve the performance of position estimation and tracking for multiple targets. Generally, radar systems suffer significantly due to clutter, when there are multiple targets or movement. However, these effects were mitigated by the detection step performed using the CFAR algorithm in our radar system. Furthermore, it is not easy to estimate the location of multiple targets using the distance values of targets detected by the radars. Therefore, we performed the position estimation for multiple targets by calculating the probability of target existence in a certain location using cumulative likelihood. By conducting simulations and experiments at the test site, we also demonstrated that the radar system designed in this work functions appropriately. In addition, the performance comparison of the proposed algorithm with the trilateration and the delay-and-sum algorithm, which is the most commonly used algorithm in the through-wall radar systems, demonstrates the validity of the proposed algorithm.

## Figures and Tables

**Table 1:** NVA6100 Specifications.

Parameter	Value
Central Frequency	1.8 GHz
Bandwidth	2.8 GHz
Average Transmission Power	−14 dBm

**Table 2:** Simulation Setup.

Parameter	Value
Position of Radar 1	(−0.49, −0.22) (m)
Position of Radar 2	(−0.14, −0.22) (m)
Position of Radar 3	(0.16, −0.22) (m)
Position of Radar 4	(0.51, −0.22) (m)
Detection rate of each radar	75%
False alarm rate of each radar	10%
Distance error of detected targets	Normal distribution with σ=0.03 (m)

**Table 3:** Experimental Scenarios.

Index	Number of Targets	Scenario
Scenario 1	1	One target standing at the coordinates (0, 6) (m)
Scenario 2	3	Three targets standing at the coordinates (1, 4.5), (−1.5, 2.5) and (0, 8) (m)
Scenario 3	5	Five targets standing at the coordinates (−4, 2), (−2, 6), (0, 9), (3, 7) and (4, 3) (m)
Scenario 4	2	Two targets standing very close each other
Scenario 5	2	Two targets moving cross along parallel paths
Scenario 6	3	Three targets moving along their respective paths
Scenario 7	2	Two targets moving cross over
Scenario 8	3	Two targets standing and one target moving
Scenario 9	1	One target moving in a circle
Scenario 10	2	One target moving in a circle and one target moving horizontally

**Table 4:** Experimental Results for the Simulations.

Scenario	Algorithm	Detection Rate (%)	False Alarm Rate (%)	$\mathrm{Position}\;\mathrm{Error}\;(m^{2})$
1	Trilateration	100	2.43	0.05
	Delay and Sum	99.86	33.95	1.22
	Proposed Algorithm	100	0.71	0.06
2	Trilateration	99.76	3.99	0.20
	Delay and Sum	98.95	44.37	2.13
	Proposed Algorithm	100	0	0.06
3	Trilateration	75.29	0.86	0.93
	Delay and Sum	94.41	57.49	7.02
	Proposed Algorithm	77.49	1.14	0.29
4	Trilateration	98.93	21.26	0.86
	Delay and Sum	54.99	0.57	6.08
	Proposed Algorithm	99.50	0	0.08
5	Trilateration	98.29	2.14	0.47
	Delay and Sum	93.15	25.11	1.24
	Proposed Algorithm	99.14	1.43	0.09
6	Trilateration	90.35	7.56	0.59
	Delay and Sum	78.22	8.70	1.00
	Proposed Algorithm	95.91	1.43	0.24
7	Trilateration	92.15	0.71	0.42
	Delay and Sum	80.39	8.27	1.05
	Proposed Algorithm	92.94	0.43	0.13
8	Trilateration	99.52	12.27	0.68
	Delay and Sum	79.89	8.56	1.22
	Proposed Algorithm	98.48	1.71	0.13
9	Trilateration	99.29	1.14	0.14
	Delay and Sum	98.43	12.13	0.59
	Proposed Algorithm	100	0.29	0.13
10	Trilateration	96.36	1.43	0.29
	Delay and Sum	93.22	25.82	1.62
	Proposed Algorithm	96.93	1.14	0.11

**Table 5:** Experimental scenarios.

Index	Scenario
Scenario 1	One person standing with a natural position at the coordinates (0, 6) (m)
Scenario 2	Three persons standing with a natural position at (−1, 4), (2, 6) and (0, 9) (m)
Scenario 3	One person walking back and forth in the radar coverage area

**Table 6:** Experimental Results for the Three Scenarios.

Scenario	Algorithm	Detection Rate (%)	False Alarm Rate (%)	$\mathrm{Position}\;\mathrm{Error}\;(m^{2})$
1	Trilateration	100	0	0.03
	Delay and Sum	100	2.18	0.57
	Proposed Algorithm	100	0	0.09
2	Trilateration	84.53	1.63	1.35
	Delay and Sum	79.17	5.90	2.35
	Proposed Algorithm	86.65	0.64	0.89
3	Trilateration	100	0	0.12
	Delay and Sum	97.59	14.82	0.49
	Proposed Algorithm	100	0	0.19
